# Toward Reservoir-on-a-Chip: Rapid Performance Evaluation of Enhanced Oil Recovery Surfactants for Carbonate Reservoirs Using a Calcite-Coated Micromodel

**DOI:** 10.1038/s41598-020-57485-x

**Published:** 2020-01-21

**Authors:** Wonjin Yun, Sehoon Chang, Daniel A. Cogswell, Shannon L. Eichmann, Ayrat Gizzatov, Gawain Thomas, Naimah Al-Hazza, Amr Abdel-Fattah, Wei Wang

**Affiliations:** 1 Aramco Services Company, Aramco Research Center–Boston, 400 Technology Square, Cambridge, Massachusetts 02139 United States; 2 Saudi Arabian Oil Company: EXPEC Advanced Research Center, Dhahran, Kingdom of Saudi Arabia; 3 Present Address: Energy Resources Engineering, Stanford University, Stanford, California 94305 United States; 4 Present Address: Department of Chemical Engineering, Massachusetts Institute of Technology, Cambridge, MA 02139 United States; 5 Present Address: Aramco Services Company: Aramco Research Center – Houston, Houston, Texas 77084 United States

**Keywords:** Energy science and technology, Nanoscience and technology

## Abstract

Enhanced oil recovery (EOR) plays a significant role in improving oil production. Tertiary EOR, including surfactant flooding, can potentially mobilize residual oil after water flooding. Prior to the field deployment, the surfactant performance must be evaluated using site-specific crude oil at reservoir conditions. Core flood experiments are common practice to evaluate surfactants for oil displacement efficiency using core samples. Core flood experiments, however, are expensive and time-consuming and do not allow for pore scale observations of fluid-fluid interactions. This work introduces the framework to evaluate the performance of EOR surfactants via a Reservoir-on-a-Chip approach, which uses microfluidic devices to mimic the oil reservoir. A unique feature of this study is the use of chemically modified micromodels such that the pore surfaces are representative of carbonate reservoir rock. To represent calcium carbonate reservoir pores, the inner channels of glass microfluidic devices were coated with thin layers of calcium carbonate nanocrystals and the surface was modified to exhibit oil-wet conditions through a crude oil aging process. During surfactant screening, oil and water phases were imaged by fluorescence microscopy to reveal the micro to macro scale mechanisms controlling surfactant-assisted oil recovery. The role of the interfacial tension (IFT) and wettability in the microfluidic device was simulated using a phase-field model and compared to laboratory results. We demonstrated the effect of low IFT at the oil-water interface and wettability alteration on surfactant-enhanced oil displacement efficiency; thus providing a time-efficient and low-cost strategy for quantitative and qualitative assessment. In addition, this framework is an effective method for pre-screening EOR surfactants for use in carbonate reservoirs prior to further core and field scale testing.

## Introduction

Crude oil is used to produce gasoline and petrochemicals that are later used to manufacture a variety of consumer goods. Therefore, oil production significantly affects the global economy and as such, improving oil recovery is a high priority^1^. Oil recovery is initiated by a geological investigation to identify oil and gas reservoirs. Once reservoirs are identified, injector and producer wells are drilled in strategic locations. During primary hydrocarbon recovery (depletion), reservoir pressure drives hydrocarbons from the reservoir to the surface through the complex pore network of the reservoir rock. In many cases, primary recovery is followed by secondary recovery where water is injected to push hydrocarbons to the producer wells. Rock properties, including wettability, capillarity, and permeability, vary and affect hydrocarbon recovery efficiency. Typically, only 30–50% of original oil in place (OOIP) is recovered through primary and secondary oil recovery processes^2^. The low recovery during the primary recovery stage has been attributed to the geophysical and geochemical complexities of oil-bearing rock in many reservoirs. Hence, the applications of tertiary Enhanced Oil Recovery (EOR) processes including the injection of chemical additives into reservoirs have gained attention as methods to promote the extraction of residual oil.

The residual oil after water-flooding is believed to be in the form of discontinuous oil ganglia trapped in the pores of reservoir rock^2,3^. Two major forces, viscous and capillary, significantly influence the microscopic movement of the trapped oil ganglia and, subsequently, oil recovery efficiency. Microscopic oil-displacement efficiency is determined by the ratio of these forces known as the capillary number ${N}_{c}=\frac{{\mu }_{j}{u}_{j}}{\sigma }$, a dimensionless number that relates the magnitudes of the capillary force and viscous forces where μ_*j*_ is the viscosity of the injected fluid, *u*_*j*_ is the Darcy velocity of the injected fluid, and *σ* is interfacial tension (IFT) between the phases. The capillary number determines whether capillary or interfacial forces dominate at the pore scale. For a capillary number around 10^−3^, the capillary and viscous forces are similar. Capillary number is usually low for slow flow reservoirs. Thus, under practical reservoir conditions the capillary number is ~10^−6^ indicating capillary-dominated flow conditions^2^. Datta *et al*.^4^ demonstrated various *N*_*c*_ (from 6 × 10^−7^ to 6 × 10^−3^) by changing only fluid velocity and directly visualized the changes in the formation and mobility of trapped non-wetting fluid ganglia within a 3D porous medium model. It was found that non-wetting fluid ganglia started to mobilize as *N*_*c*_ is increased above a threshold value of 2 × 10^−4^.

EOR methods are used to mobilize residual oil and bring it to the surface^5–8^. Chemical EOR methods use specially selected injectants in the water phase to extract unrecovered hydrocarbons. These injectants, such as water/seawater and chemical solutions, have been flooded as secondary and tertiary EOR methods, respectively. Surfactants are common additives for chemical EOR methods as they alter wettability and reduce the IFT between oil and water^3,9–15^. The most effective surfactant formulations reduce the IFT between the residual oil ganglia/water interface and increase the capillary number to 10^−2^–10^−4^ ^2,3^. Given that a high IFT is the main cause of trapped ganglion in porous media, reducing the IFT is an effective method to mobilize trapped oil drops^9,16^.

Applying chemical EOR processes can significantly improve oil recovery but proper additive selection is required^3^. The effects of the porous media structure and chemistry spans length scales from the pore, to the core, to the reservoir scales. Thus multiscale approach to evaluate EOR chemical materials are necessary to understand their transport, phase behaviors, and surface chemistry. Pore-scale observations may be employed in concert with core-scale observations to understand the coupled mechanisms that exist over a wide range of scales. At the field-scale, greater than a meter, numerical models are validated and calibrated to laboratory data at the pore and core scale^17,18^. In this fashion, the transition from pore to field scale can be made more rigorously with the aid of a computer model to extrapolate microfluidic chip results to reservoir conditions. Thus, prior to testing additives at the field scale, it is necessary to evaluate chemical performance in the laboratory under the conditions similar to specific reservoir, such as high pressure, high temperature, and high salinity. These tests, however, are time-consuming and expensive and, screening a large library of chemicals at reservoir conditions is not possible in all cases. In recent years, however, the ability to select low-cost, high-performance surfactants for a wide range of crude oils under a wide range of reservoir conditions has improved dramatically. Yang *et al*.^7^, for instance, presented a macro-scale phase behavior approach for developing surfactant formulations based on a wide variety of fluid additives such as, surfactants, co-surfactants, co-solvents, alkali, polymers, and electrolytes.

Carbonate reservoirs, such as those present in the Middle East, contain considerable micro-porosity^19^. The complexity and disorder of the pore structure provides an ongoing challenge to understanding the distribution of fluids and the fluid flow behavior^20,21^. Alyafei and Blunt^22^ revealed the complex effect of surface properties, such as wettability on the relationship between the residual oil saturation and the initial oil saturation in carbonates. Furthermore, Lifton^1^ emphasized that the characteristic length-scale of the pores in oil-bearing rocks is the micro-scale. As a result, EOR chemical performance optimization should be coupled with the observation of micro-scale phenomena where capillary forces and surface tension overcome the effects of gravity and inertia and become dominant.

Microfluidic devices, so-called micromodels or “Reservoir-on-a-chip” (ROC), are quasi-2D representations of porous media that allow direct visualization of pore-scale fluid flow^23–26^. Pore-scale observations using silicon- or glass-based micromodels have been widely used to understand multiphase flow, oil-water-solid interactions, and dynamics of microemulsions in many EOR studies^10,11,27–31^. Wang *et al*.^32^ recently presented a visual investigation of the gas-water flow mechanism in the micromodel mimicking the fracture-cavity carbonate reservoirs. In addition, improvements have made it possible for micromodels to represent realistic field conditions such as wettability, high temperature, high pressure, and surface geometry, therefore bringing a better understanding of fluid flow behavior at the micro-scale^33–40^. Furthermore, Marchand *et al*.^41^ discussed the challenges associated with drawing robust quantitative oil recovery conclusions from micromodel datasets which include data dispersion and experimental repeatability verification. The authors provided a guideline for the acceptable number of repetitions of micromodel experiments needed to derive the quantitative conclusions of oil phase recovery. Another study demonstrated that the oil recovery trends from micro-scale chip data can be correlated to core-scale water flooding experiments^23^. In addition, He *et al*.^42^ demonstrated fundamental differences between two surfactant types using a ROC approach to screen surfactants for hydraulic fracturing application in the Eagle Ford Shale.

These previous studies were highly effective but do not capture the effect of surface chemistry at the pore scale on the recovery process in carbonate reservoirs. Toward the Reservoir-on-a-chip, we firstly developed a nanofabrication process (*in situ* CaCO_3_ growth) to generate homogeneous calcite surface coverage in microfluidic devices where the carbonate chemistry is now resprented^43^. Here, we further demonstrate a carbonate ROC microfluidic approach for fast and cost-effective pre-screening of chemical EOR additives to identify the best performer as a top candidate for core scale oil recovery tests and later field tests. This study highlights the relevance of the capillary processes and fluid movement inside and across the entire domain of porous materials to evaluate non-emulsifying surfactant-assisted oil recovery processes. The excellent optical transparency of the CaCO_3_-coated microfluidic device allows the combination of optical fluorescence microscopy together with image processing to present microfluidics as a powerful tool to aid in the study of chemical EOR formulations. Simulation results are also included where the exact chip geometry is used with a phase-field model to demonstrate the effects of changing injected fluid and surface wetting properties on oil recovery which confirms our experimental results.

## Experiment and Methodology

### Fluids

Water was deionized with a Milli-Q purification system. Synthetic seawater was prepared by dissolving 41.042 g NaCl, 2.385 g CaCl_2_ ∙ 2H_2_O, 17.645 g MgCl_2_ ∙ 6H_2_O, 6.343 g Na_2_SO_4_, and 0.615 g NaHCO_3_ into 1 L of de-ionized (DI) water. Next, 200 mL of 10^−3^ M fluorescein stock solution was prepared by dissolving 6.64 × 10^−2^ g fluorescein (Thermo Fischer Scientific) in the mixture of 190 mL DI water, 9 mL ethanol, and 1 mL of 1 M NaOH. 10^−4^ M fluorescein-dyed in seawater was prepared by diluting 10^−3^ M of fluorescein stock solution with seawater. Crude oil was used as an oil phase, which is unfiltered and composed of 33.1% saturates, 47.3% aromatics, 8.7% resins (polars I), and 10.9% asphaltenes (polars II) according to SARA (saturate, aromatic, resin, and asphaltene) analysis. Solutions of 0.2 wt% EOR surfactant, labeled as Sample A and Sample B, were prepared. Sample A was a mixture of alkylbenzene sulfonate and cocamidopropyl hydroxysultaine as described previously^44,45^. Sample B is a commercially available surfactant, cocamidopropyl hydroxysultaine (Petrostep - SB, 43 wt% active, Stepan Company, Northfield, IL) and was prepared by diluting the stock solution with seawater to achieve 0.2 wt%. Surfactant fluid properties are presented in Table 1.

**Table 1:** Fluid properties and fluid-surface properties at room temperature.

Surfactant formulation	ρ_f_ (g/cm^3^)	µ_f_ (mPa^*^s)	ϴ_o,aged_ (°)**	IFT_eq_^***^ (mN/m)	N_c_^*^
Sample A	1.034	1.013	88 ± 4	0.005 ± 0.003	2.86E-03
Sample B	1.035	1.005	144 ± 14	0.357 ± 0.046	3.97E-05

^*^N_c_ = µ_f_ *u*_*j*_/σ, *u*_*j*_ = 2.4 ft/day.

^**^Images from Fig. S4.

^***^The IFT_eq_ was measured with synthetic seawater and crude oil using a Krüss Spinning Drop tensiometer.

### Microfluidic chips

**Figure 1:**
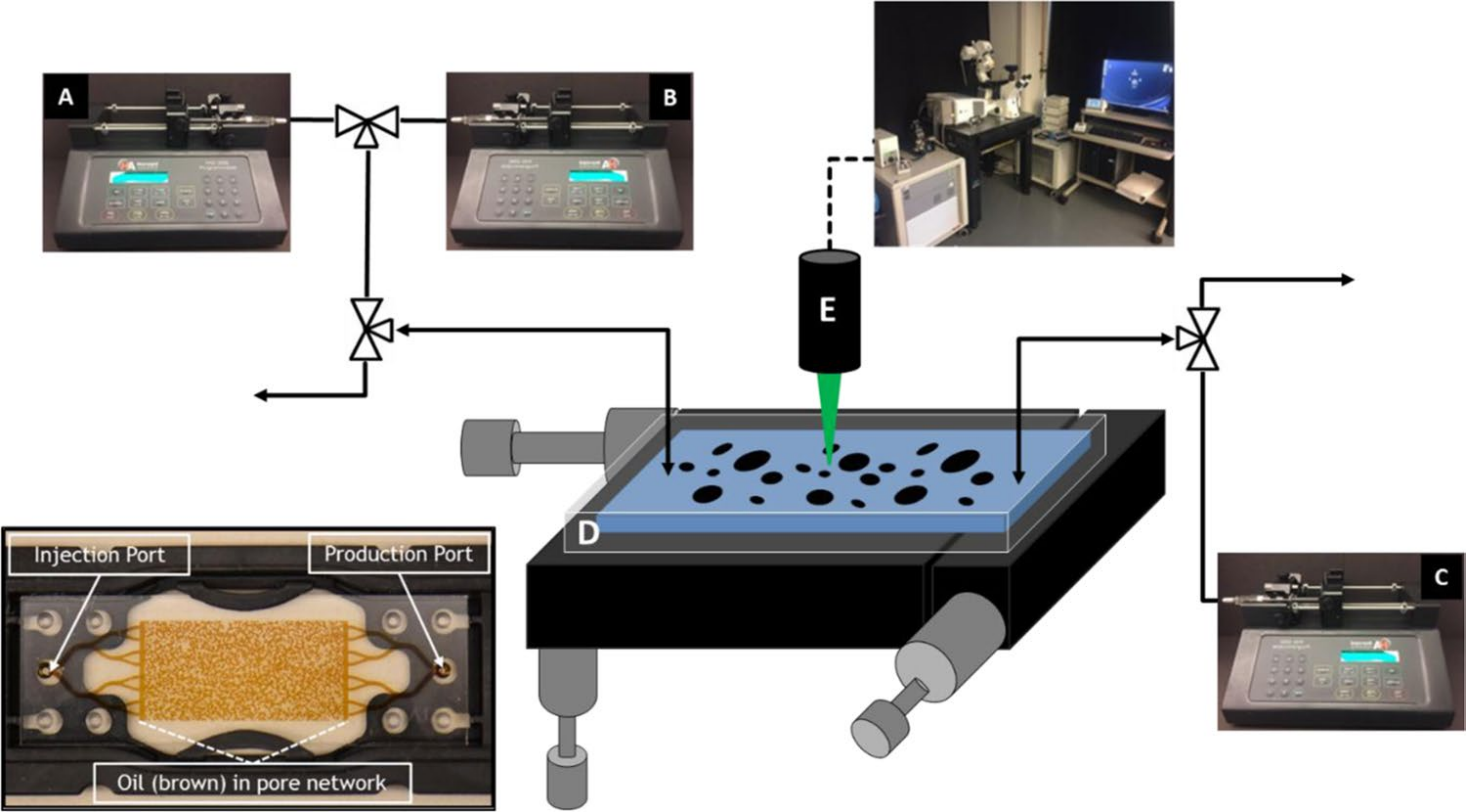
Experimental setup showing the seawater (**A**), surfactant (**B**), and crude oil (**C**) injection pumps and the micromodel (**D**). Images were obtained via Zeiss Observer Z1 inverted microscope (**E**). Inset: an image of the micromodel (**D**) indemnifying the injection and production ports and the pore network filled with crude oil.

**Figure 2:**
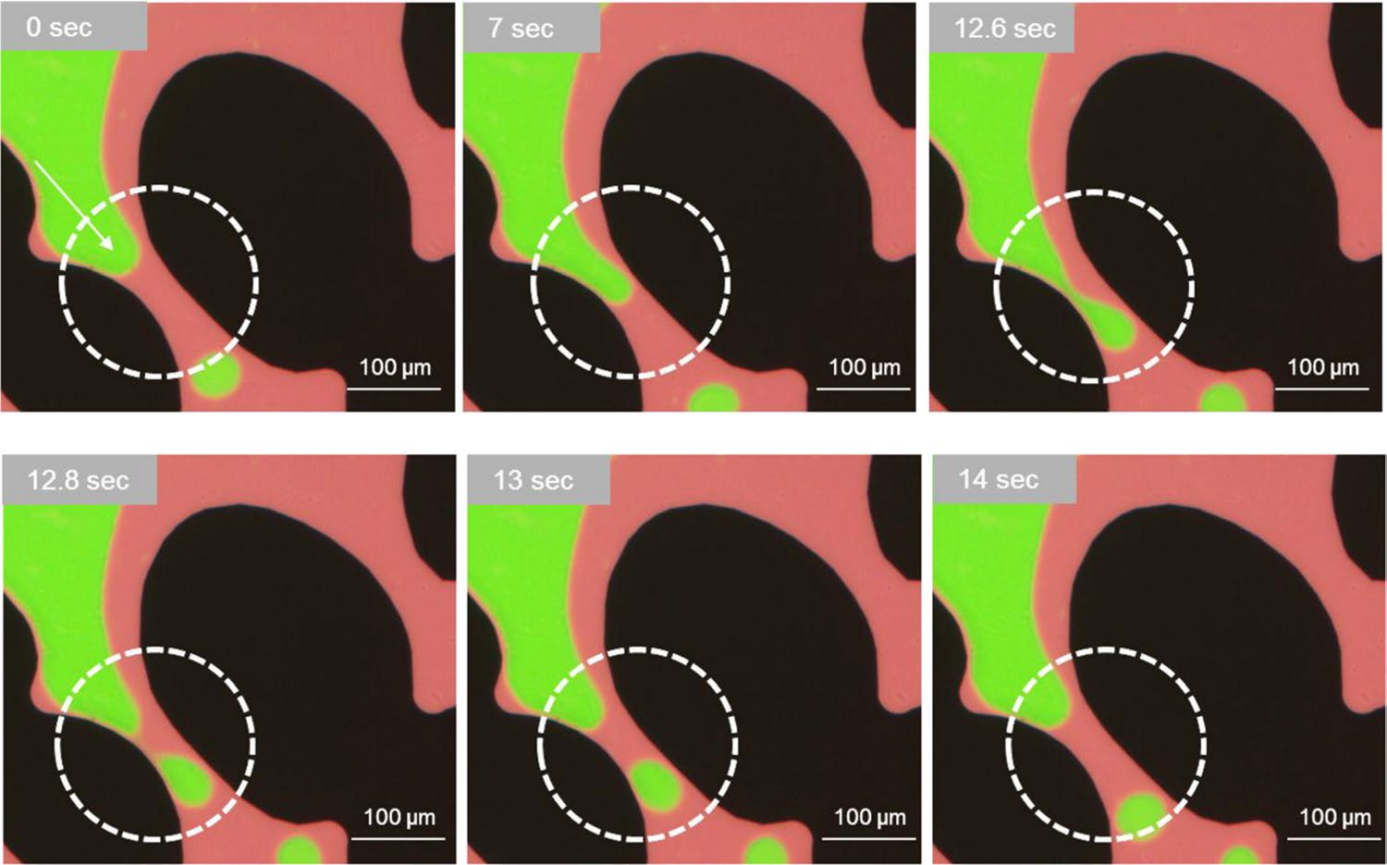
Pore level observation showing a thin layer of oil (red) remaining on the pore walls, preventing water (non-wetting phase) from contacting the rock surface. Snap-off events at the single-constricted pore channel (dashed-circles) throughout the oil-phase injection. (0 sec to 7 sec): non-wetting phase (water in green) penetrates as capillary pressure exceeds the capillary entry pressure. (12.6 sec to 12.8 sec): water-front enters the wider pore body in the downstream of the throat. Reduced capillary pressure causes snap-off in the upstream throat. (14 sec): waterfront recedes to its initial shape.

**Figure 3:**
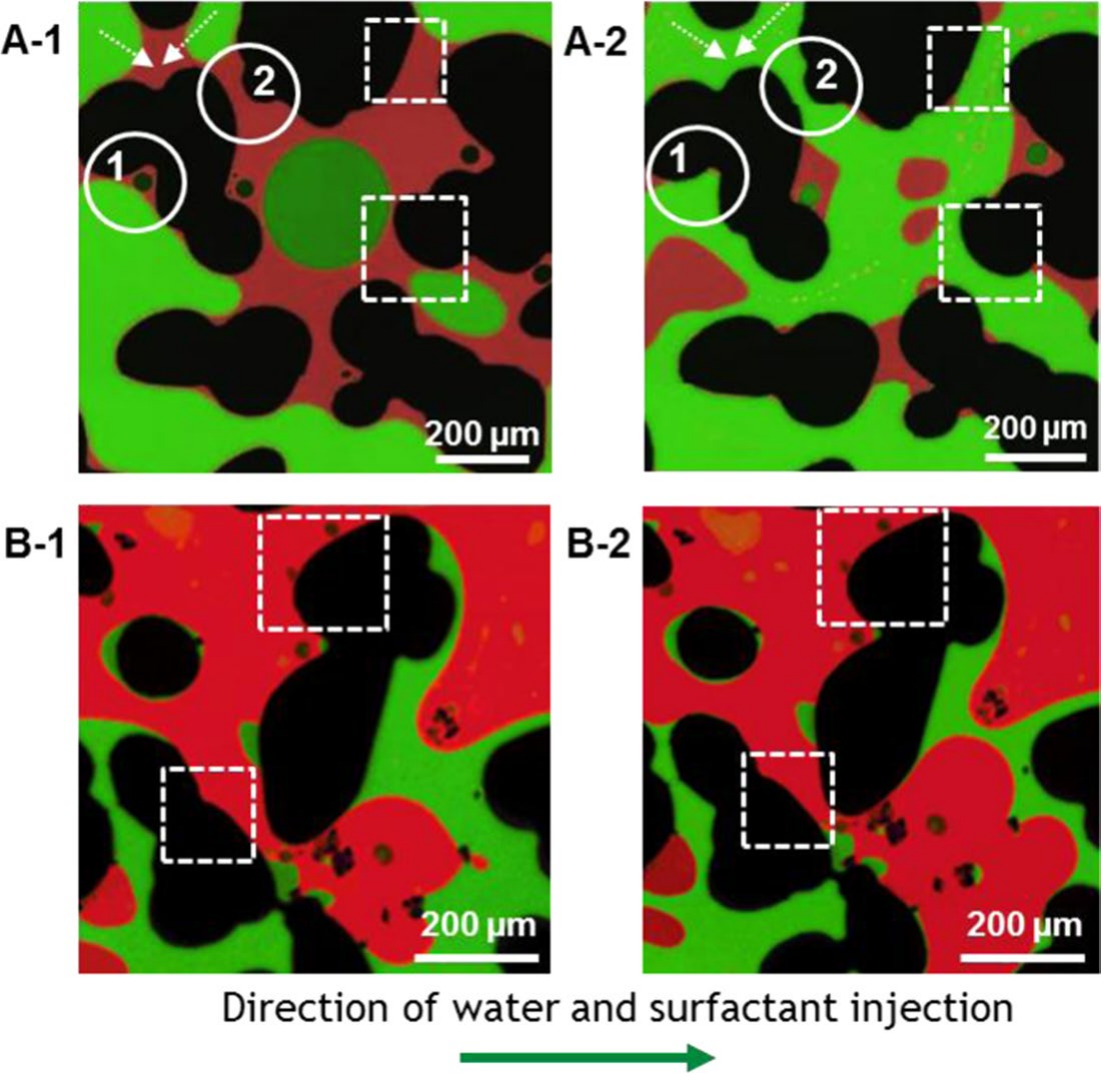
Microscopic images of pore-scale wettability alteration before and after 1 PV injection of Sample A and Sample B (defending fluid: red) and water phase (invading fluid: green). (**A-1** and **B-1**): pores exhibit partially oil-wet conditions with surface affinity (dashed square) to the oil phase before Sample injection. (**A-1**→**A-2**): Sample A induced wettability alteration showing water in contact with grain wall (dashed-square) and cooperative pore filling (dashed arrow) of invading fluid (water). Circle 1 shows recovery of trapped residual oil by water invasion into capillary end. Circle 2 demonstrates water enters pore throat without snap-off due to the reduction of oil-wet characteristic. (**B-1**→**B-2**): Sample B does not result in significant wettability alteration (dashed-square) and does not mobilize much trapped oil.

EOR microfluidic chips were purchased from Micronit Microtechnologies (Netherlands) and used as a microfluidic platform for the surfactant-screening test (Fig. 1). The EOR vuggy chip from Micronit has a porous matrix 20 mm long by 10 mm wide and 20 µm etching-depth. Prior to the porous matrix, inlet and outlet channels act as flow distribution fractures that are 500 µm wide. The pore volume is 2.4 µL and matrix porosity is ~60%. Using our recently developed method^43^, the surface of the microfluidic channels has been fully converted from the original silica to calcium carbonate (CaCO_3_) by *in-situ* growth of a 1–2 µm thin layer of CaCO_3_ nanocrystals on the surface. Since temperature, water composition and buffering capacity may have a great impact on the kinetics of mineral^17^, we performed stability test on the coated calcite nanocrystal layers and no dissolution was observed at our experimental condition. To change the wettability of the CaCO_3_ surface, the micromodel was filled with crude oil and aged for >48 hrs at 95 °C prior to water saturation to create an oil-to-mixed wettability state (see Figs. 2 and 3). Then the absolute permeability (*k*) of the carbonate micromodel was calculated using Darcy’s law, $k=-[\frac{q{\mu }_{w}}{A}][\frac{L}{\Delta P}]$, where *A* is the cross sectional area (product of width, 1 cm, and etching depth (H = 20 µm)), Δ*P* is the pressure drop in atm, *μ*_*w*_ is the viscosity of water in cp, *q* is the volumetric flow rate in mL/sec, *L* is the length (2 cm), and *k* is the absolute permeability in Darcy. The steady-state pressure drop across the micromodel was measured at different seawater flow rates (0.001–0.02 mL/min). Using the dimensions of the micromodel, known constants, and measured variables, the product of q and μ_w_, divided by A versus ΔP divided by L was plotted, and the slope, which is equal to k, was calculated. The permeability, k, for the micromodels used in this experiment was 1.97 Darcy, which is relatively high for carbonate rock but not unreasonable given the size of pores in this micromodel (Fig. S1).

### Fluid characterization

The viscosity and density of the solutions were measured using an Anton-Paar SVM-3001 Viscometer. Values were recorded at 25 °C. The IFT was measured using a Krüss Spinning Drop densiometer. A total of 100 measurements, sampling a minimum of 3 drops, were collected over a period of 30 minutes at 25 °C. At each measurement, the drop shape was fitted with the Young-Laplace equation to determine the interfacial tension, and the values were averaged.

### Injection procedure

**Figure 4:**
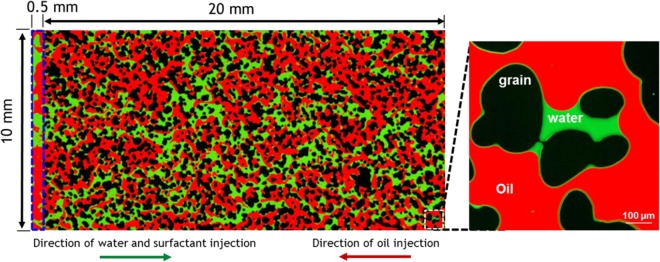
Single fluorescence image (right) of the EOR vuggy microfluidic chip and a fully stitched fluorescence image containing >500 tiles (left). The dimensions of the porous matrix are 20 mm by 10 mm with a 20 µm depth. Flow distribution channel (blue rectangle) has a width of 0.5 mm.

To establish an initial condition of the experiment, the pore space of the oil-aged micromodel was fully saturated with 10^−4^ M fluorescein dyed seawater using the syringe pump (flow direction shown in Fig. 4). The dyed seawater provided a green fluorescence used to distinguish the oil and water phases during imaging and image processing. After the initial water injection from pump A in Fig. 1, crude oil was continuously injected (0.1 μL/min) into the seawater saturated micromodel using pump C in Fig. 1. Once the injected oil phase breaks through into opposite-side distribution fracture (shown in Fig. 4), the oil injection was suspended and the image analysis determined the residual water saturation (S_wr_) and original oil-in-place (initial oil saturation = S_oi_).

At the residual water saturation (S_wr_), a forced water imbibition experiment, so-called “secondary water flood”, was conducted. A 10^−4^ M fluorescein dyed seawater solution was injected from pump A at a volumetric flow rate of 0.1 µL/min to extract the oil phase from the micromodel, which superficial velocity (Darcy velocity) is 2.4 ft/day at capillary number (N_c_) 4.93 × 10^−7^. The images obtained during the secondary waterflood were analyzed to monitor the decrease in oil saturation (S_o_).

When oil production rate becomes significantly low, a forced surfactant solution imbibition experiment, a so-called “tertiary surfactant flood”, was conducted to extract additional oil phase from the micromodel. Specifically, 1 pore volume (PV) of surfactant solution was injected into the micromodel using channel A at a volumetric flow rate of 0.1 µL/min (superficial velocity is 2.4 ft/day). After 1PV of surfactant injection, seawater flooding was continued until the oil saturation in the micromodel had plateaued and no more oil was displaced.

### Fluorescence microscopy and image processing

The images of fluids in the micromodels were collected using a widefield fluorescence microscope (Observer. Z1, Zeiss) with a 10x microscope objective and a color camera. The fluorescence images were collected using a filter cube with the combination of excitation (390–422 nm, 484–501 nm, 549–573 nm) and emission (448–472 nm, 512–538 nm, 585–631 nm) bands. Each full-chip image (Fig. 4) represents >500 tile images (36,000 pixels × 19,000 pixels, 0.64 µm/pixel) that were stitched together using the tile feature of the ZenBlue software by Zeiss.

Images were processed using MATLAB and the Image Processing Toolbox. For each image, the region of interest (ROI) was selected to include only the porous matrix portion of the image using the ROIPOLY function in MATLAB. The ROI section of image was then separated into red, green, and blue channels from the full color RGB image. Next, the GRAYTHRESH function in MATLAB was used to obtain the threshold for the oil (red channel) and aqueous (green channel) phases in the image where Otsu^46^ thresholding was used. Finally, the number of pixels above the threshold in the R channel (R) were counted as the oil phase and the number of pixels above the threshold in the G channel (G) were labeled as the aqueous phase (i.e. Sample A or Sample B).

The porosity of the chips was recorded based on the counted pixels as shown in Eq. 1. The standard deviation in the porosity for each run was less than 3% and this was used as the method to detect changes in the amount of total fluid filling the channel.

1 $$\phi =100\ast (\frac{R+G}{Are{a}_{ROI}})$$

The oil saturation (S_o_) was calculated as shown in Eq. 2 and the water saturation was calculated as %S_w_ = 100 − %S_o_.

2 $$ \% {{S}}_{o}=100\ast (\frac{R}{R+G})$$

Next, the percentage of the original oil in place (OOIP) that has been recovered was calculated as the difference in the amount of oil in the chip for each image from that of the initial image before seawater injection began (Eq. 3).

3 $$ \% OOIP\,Recovered=100\ast (1-\frac{{S}_{o}}{{S}_{oi}})$$

Finally, the percentage of incremental oil recovered (% Incremental Recovery) was calculated as the difference between the percentage of OOIP recovered at the end of the experiment and that after the initial seawater flush. Rather than using only the data from the last image in both of these cases, the average of the last few images where the recovery had reached a plateau was used. This method reduced the likelihood that artifacts, such as oil release from the inlet channels prior to the porous matrix or bubble encroachment at the end of the experiment, would significantly alter the % Incremental Recovery.

### Phase-field model of two-phase flow at the pore scale

A phase-field model was developed for simulating oil/water displacement through arbitrarily complex pore structures. The model was recently applied to pore-scale two-phase flow in a radial Hele-Shaw cell under a variety of wettability conditions, and found to capture many of the features of the displacements^47^. The model is based on the Cahn-Hilliard-Hele-Shaw system, which describes depth-averaged fluid flow between two parallel plates (*i*.*e*. a Hele-Shaw cell) and accounts for interfacial tension between the two fluids. The solid pore walls were treated with no-penetration boundary conditions that enforce zero velocity normal to the surface, but do not enforce a no slip condition. This is a reasonable assumption for Hele-Shaw cells, where a no-slip condition is only valid close to a wall, a distance on the order of the separation between the parallel plates (20um). An additional variational boundary condition was introduced to account for the contact angle of the fluids at the pore walls. More details of the model are provided in the supplementary information (SI).

## Results and Discussion

Screening of two different EOR surfactant formulations, their oil recovery efficiency, and the pore-level observation of the displacement of wetting fluid phase (oil) by non-wetting fluid phase (seawater) in the CaCO_3_ coated EOR chip have been demonstrated. Specifically, the microscopic images of the micromodel pore network with initial oil and seawater saturation were taken before and after water and surfactant injection experiments. Fluorescence microscopic images were used to evaluate the efficacy of water flood and surfactant flood for extraction of hydrocarbon from the rock pore geometry. The values of incremental oil recovery for each run of the experiment were calculated and compared for the quantitative determination of the efficiencies of the surfactant formulation between Sample A and Sample B.

### Surfactant performance

**Figure 5:**
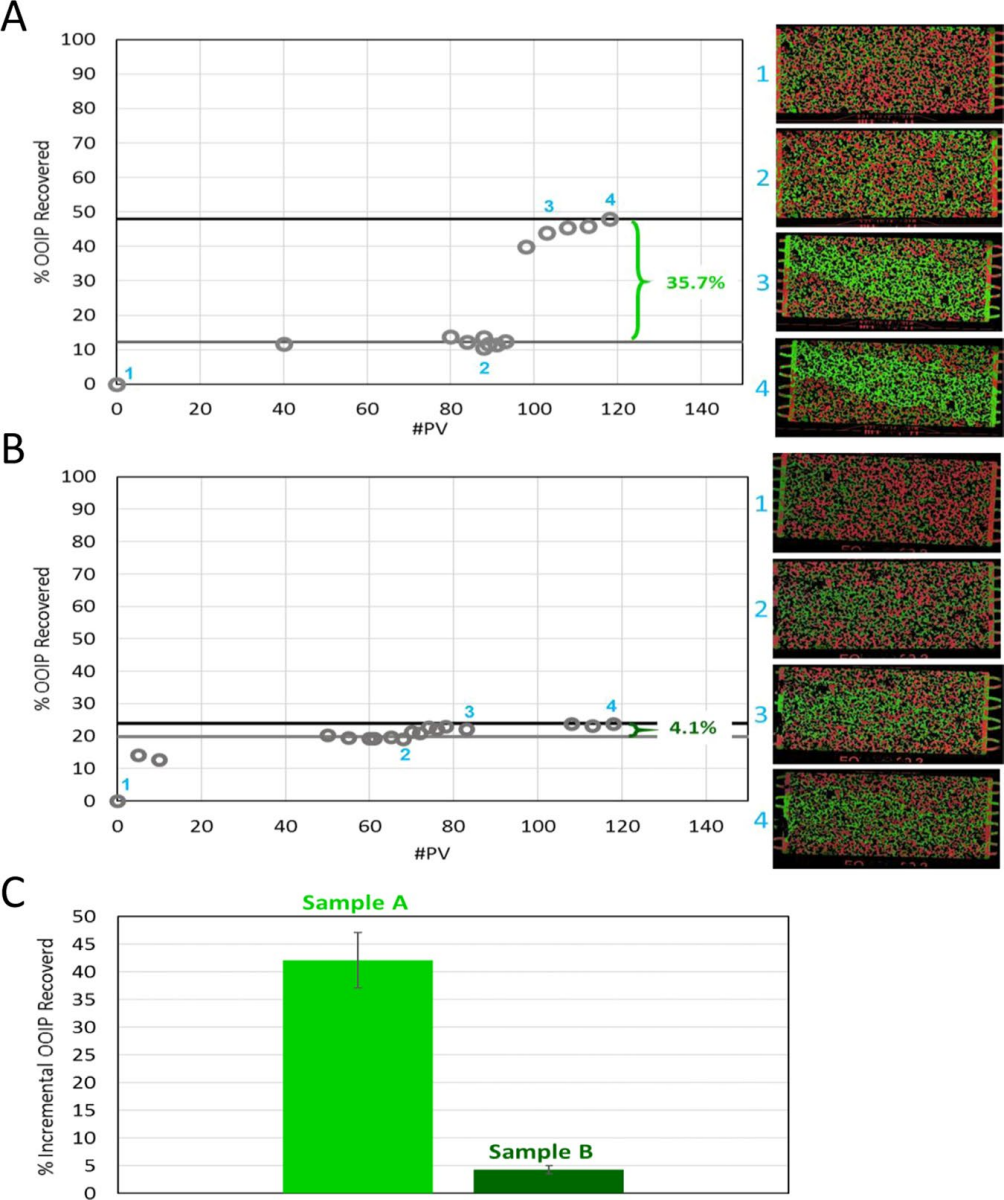
Cumulative oil recovery (% OOIP) plotted against cumulative pore volume (PV) injected for (**A**) Sample A and (**B**) Sample B. Incremental oil recovery after injection of 1 PV of surfactant was 35.7% for Sample A and 4.1% for Sample B. (**C**) Average incremental recovery from three runs of Sample A versus Sample B. Sample A recovered ~10× more oil than Sample B.

**Figure 6:**
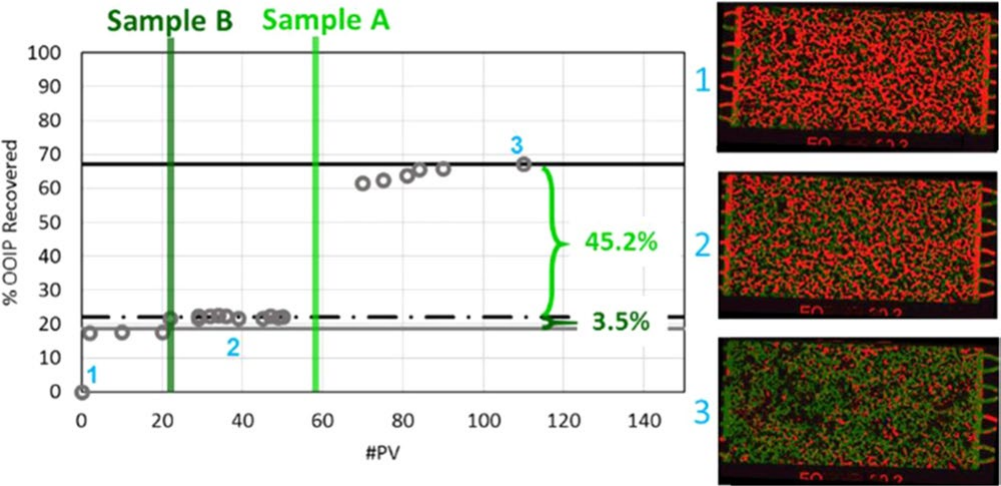
Cumulative oil recovery (%OOIP) from a consecutive injection series where 1 PV of Sample B was followed by seawater and then by 1 PV of Sample A then by seawater. Incremental oil recovery after injection of 1 PV of Sample B was 3.5% and the subsequent 1 PV injection of Sample A followed by seawater resulted in 45.2% incremental recovery.

Here, the oil recovery efficacy of surfactant formulations, in-house produced Sample A and commercial available Sample B, with different fluid properties (Table 1) were evaluated with the CaCO_3_-modified EOR chip with a random porous network. Figure 5 shows representative data for Sample A and B used to compare the efficacy of each in terms of enhancing the cumulative oil recovery (% OOIP). Specifically, the cumulative % OOIP recovered is plotted according to the cumulative pore volume injected, including initial seawater flood, surfactant injection, and post-injection of seawater, for Sample A in Fig. 5A and Sample B in Fig. 5B. As demonstrated by the contact angle measurements, wettability alteration during crude oil aging converted the CaCO_3_ from water-wet to more oil-wet. As such, the injected seawater during the initial seawater flood did not effectively displace oil, resulting in low recovery (10 ~ 20% OOIP) after the water flooding. This is not the case after surfactant injection. For both surfactant formulations, surfactant flooding mobilized additional oil. In the case shown in Fig. 5, Sample A performed significantly better at improving incremental oil recovery. After the 1PV injection of Sample A an additional 35.7% OOIP was recovered, while Sample B recovered only 4.1% OOIP more than water flooding. Each surfactant formulation was tested three times for repeatability (Fig. S2) and the average additional %OOIP recovered for each is compared in Fig. 5C. The data show that Sample A provided significantly greater incremental oil recovery, ~40% OOIP, when compared to the recovery using Sample B, ~4% OOIP, a relative performance difference of 10× . The results demonstrate that the 10x performance improvement measured for Sample A is statistically significant without performing the number of analyses suggested by Marchand *et al*.^41^. For further verification of the performance difference, an additional experiment was carried out (Fig. 6). Here, after the initial seawater flood, 1 PV of Sample B was injected followed by additional seawater injection until the additional recovery from Sample B ceased. This was then followed by a 1 PV injection of Sample A followed by additional seawater injection until recovery once again ceased. Again, Sample B produced 3.5% additional recovery, while a subsequent injection of Sample A produced 45.2% of incremental oil recovery which is consistent with the results in Fig. 5.

The greater incremental oil recovery for Sample A over Sample B can be explained by a larger increase in capillary number *N*_c_ for Sample A and Sample B due to significant differences in the IFT (Table 1). The oil-water IFT_eq_ for Sample A is 0.005 ± 0.003 mN/m and for Sample B is 0.357 ± 0.046 mN/m. Due to the reduction of oil-water IFT_eq_ by Sample A and B, the capillary number (***N***_*c*_) for Sample A injection at a constant injection rate would increase from 4.93 × 10^−7^ to 2.86 × 10^−3^ while it would only increase to 3.97 × 10^−5^ for Sample B. The two orders of magnitude difference in ***N***_*c*_ between Sample A and Sample B would indicate that with Sample A the viscous forces are more dominant when compared to Sample B, leading to better recovery with the former.

### Pore- to macro-scale observation

The mechanisms of wettability alteration by crude oil have been previously studied and highlight the interactions between the oil and rock surface where asphaltenes in oil precipitate on the rock surface^48^. The amount of asphaltene precipitation and the ability to alter the wettability depend on the types of crude oil and their ability to dissolve their asphaltenes. For these CaCO_3_-coated chips, contact angle measurements on calcite-coated glass slides were used to confirm that aging the surface in crude oil resulted in an oil-wet surface. The contact angle of water on the crude oil-aged calcite-coated glass surface was reported as θ = 100° and of crude oil as θ = 24° in our previous study^43^. The pore scale observations for the crude oil aged CaCO_3_ coated micromodel confirms that the CaCO_3_ surface of the chip shows from oil- to mixed-wettability inside the channels as presented in Fig. 2, where a snap-off occurs as the non-wetting phase of seawater invades an oil-filled pore with a narrow pore throat and wide pore body. Figure 2 illustrates the real-time pore-scale observation of the snap-off process where a thin layer of interstitial oil (red) is a continuous wetting phase remaining on the pore walls due to the oil-wet calcite-coated surface, while water (non-wetting phase) repeatedly snaps off as it flows through the pore throat.

Reservoir wettability has a significant effect on oil recovery, therefore both favorable alteration of reservoir wettability and reduction of IFT help mobilize residual oil and improves oil production^9,16,49^. Figure 3 demonstrates wettability alteration before and after a 1 PV injection of Sample A and Sample B into crude oil and water-filled pores of a crude oil aged CaCO_3_-coated chip. Figure 3A-1,B-1 are single areas in the chip before Sample A and Sample B injection, respectively, that demonstrate the initial wettability of the aged chip and Fig. 3A-2,B-2 show the same areas after surfactant injection. Visual comparison of Fig. 3A-1 to A-2 at the grain boundaries shows that after the Sample A injection several oil-wet domains are converted to water-wet where the overall oil-wetness has been reduced. Cieplak and Robbins^50^ first proposed the concept of a critical contact angle θ_c_ and its dependence on porosity to alter the invading fluid pattern as a function of contact angle θ. It has been reported that the strong affinity of the displacing phase for the grain surface leads to uniform sweep and forms a remarkably compact displacement pattern. Thus this is due to cooperative pore filling, in which two or more neighboring menisci overlap and merge into a new, stable meniscus^50,51^. In Fig. 3A-1,A-2, Sample A creates more compact displacement via cooperative pore filling of invading fluid without snap-off at the pore throat because Sample A decreases the contact angle below the critical contact angle. This observation is analogous to the experimental investigations by Zhao *et al*.^51^, where they used a patterned microfluidic device with lower porosity (ϕ = 0.45) than our model (ϕ = 0.60) and systematically varied the wettability. They showed a compact displacement pattern of silicone oil by water at a contact angle of 60° which is below the critical contact angle range of 120° < θ_c_ < 150° and ***N***_*c*_ **(**2.9 × 10^−3^). For the Sample B (Fig. 3B-1,B-2) however, wettability alteration is less apparent. For example the wettability of the oil wet surface, shown in Fig. 3B-1 did not change considerably when compared to Fig. 3B-2 obtained after Sample B injection. These observations suggest that Sample A reduces IFT and produces larger wettability alteration of the oil-wet calcite surface than Sample B; thus the oil displacement with Sample A created a regime of cooperative-filling rather than a regime of corner-flow. The two orders of magnitude larger ***N***_*c*_ shown in Table 1 and the observed wettability alteration with Sample A compared to Sample B supports the 10x improvement of oil displacement efficiency for Sample A.

A series of stitched microscopic images of oil-wet CaCO_3_ treated micromodel surface along the entire flow distribution fracture and pore network near the inlet port have been collected and shown in Fig. S3. Figure S3A shows the profile of the micromodel filled with crude oil and seawater. Fig. S3B is the image at the same location after 20 PV of seawater has been injected at a constant rate of 0.1 µL/min during secondary water-flood. Here, the solid arrow indicates an oil domain that is displaced due to injection as the water phase enters the network through flow path. Additionally the yellow-dashed rectangle in Fig. S3B identifies a stagnant residual oil bank in the fracture. The large amount of residual oil in the yellow rectangle forms continuous ganglia bridged by large oil-wet CaCO_3_ crystals. Rigid oil ganglia created the entry capillary pressure between CaCO_3_ structures and induced fingering (denoted as solid-arrow) during the water-flood. The results of the secondary water-flood imply that the residual oil phases require tertiary or enhanced recovery methods to overcome the capillary forces. Fig. S3C is the image after 1 PV of tertiary surfactant flood (Sample A) and 50 PV of additional water-flood at a constant rate of 0.1 µL/min. Tertiary surfactant flood breaks up the oil ganglia and provides the additional flow paths (denoted by dashed-arrows in Fig. S3C) for seawater, thus increasing the oil displacement efficiency (yellow-dashed rectangle). Here, the breakup of the ganglia and subsequent improved oil recovery can be attributed to the reduced IFT and increased ***N***_*c*_ by Sample A to a viscous dominant flow regime. These conclusions are consistent with the observation from Datta *et al*.^4^, where IFT reduction-driven change of ***N***_*c*_ activated the breakup of trapped oil ganglia to enhance oil mobility and improve oil removal from the medium.

### EOR microchip simulation results

To better understand the two-phase displacement of the EOR process, oil recovery experiments were simulated using a phase-field model developed for Hele-Shaw flow^52–55^, which is flow through the thin gap between two parallel glass plates. Phase-field models rigorously capture the Laplace pressure jump at curved interfaces due to IFT and the resulting interfacial movement without the need to track explicit boundaries. Beginning with a model previously developed to stabilize numeric Darcy flow simulations^56^, extensions were introduced to treat two-phase flow through arbitrarily complicated pore structures. An appropriate mixture viscosity was employed to account for viscosity contrast between the phases, and a contact angle boundary condition at the pore walls was introduced^57^. Thus the model can be used to study the role of interfacial tension, contact angles, fluid viscosity, pore structure, and injection rates on two-phase displacement in thin-gap microfluidic chip.

**Figure 7:**
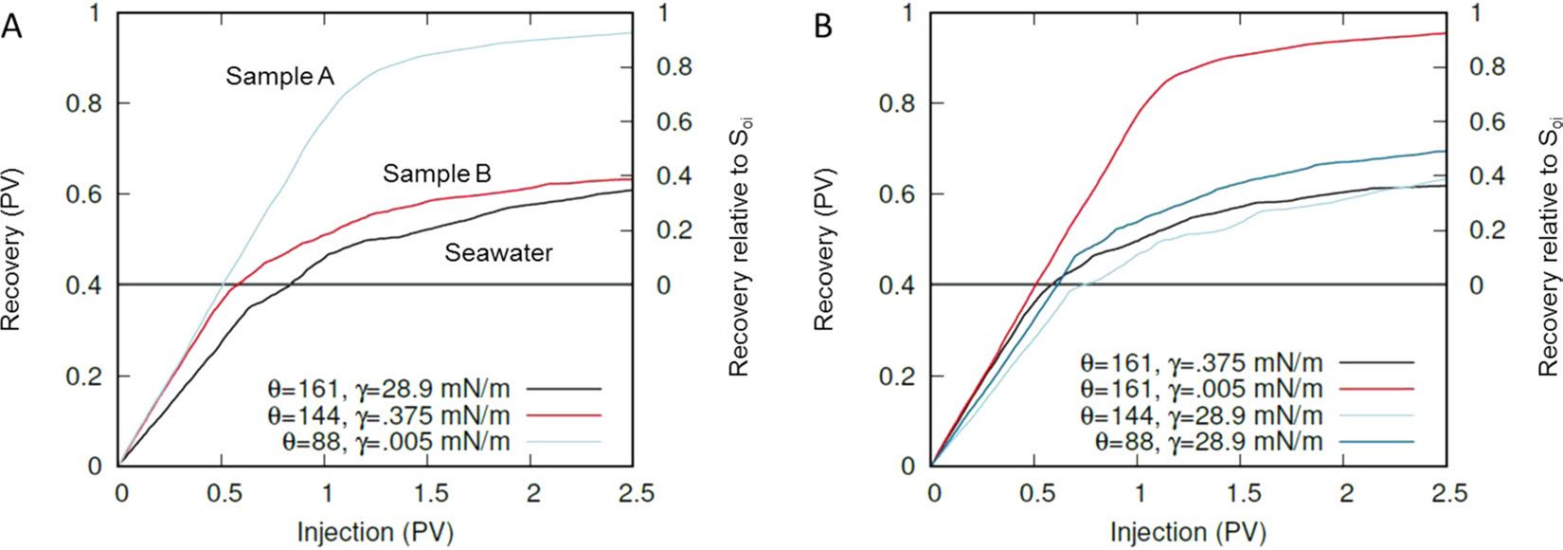
Simulated oil recovery from the EOR microchip as a function of the pore volumes of injected water for different interfacial tensions and contact angles. The Y-axis of the plots has been rescaled to show oil recovery relative to the experimental initial condition (S_oi_ = 60%) for easier comparison with experimental results. (**A**) Wettability and IFT chosen to match experiment. (**B**) Simulation of different combinations of IFT and contact angle reveals the significant effect of low IFT on improving oil recovery.

A image of the experimental chip geometry was used to generate the pore structure for simulations. The simulated oil recovery from the EOR chip during water injection is presented in Fig. 7A for the experimentally measured surfactant formulation properties, IFTs, and aged contact angles (Table 1). The simulations used fully oil saturated pores (S_oi_sim_ = 100%) as an initial condition; however, the experiments started with an initial oil saturation (S_oi_) of 60%. Hence, the right axes in Fig. 7 have been appropriately rescaled to allow for comparison of the simulated and experimental oil recoveries. Oil recoveries of 34.7%, 92.6% and 38.7%, relative to 60% of S_oi_sim_ were calculated for the seawater, Sample A, and Sample B, respectively. In this simulation, the predicted incremental recovery for Sample A after seawater was 57.9% and was only 4.0% for Sample B. This represents a predicted ~14× better improvement in oil recovery with Sample A over Sample B which is consistent with the ~10× relative difference measured experimentally.

**Figure 8:**
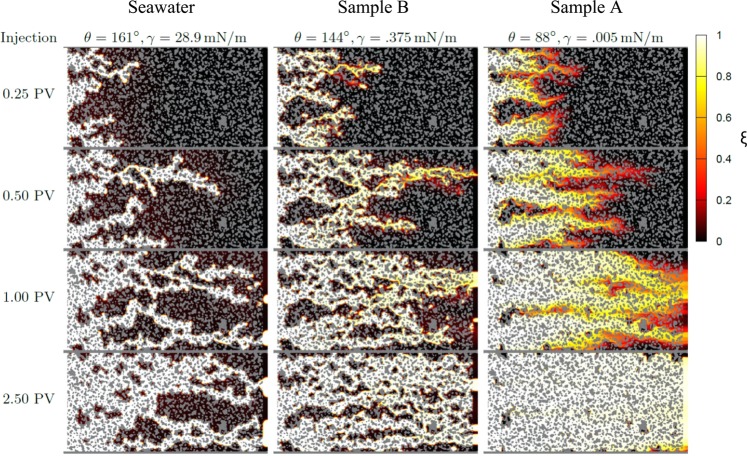
Snapshots of the EOR microchip simulation for interfacial tensions and contact angles corresponding to Fig. 7A. The solid pore walls are gray, and ξ is the depth-averaged volume fraction of water in the pores.

Simulation progression images of the oil-wet pore space are presented in Fig. 8 to demonstrate the effects of wettability alteration and IFT reduction on the oil saturation and flow path. Seawater alone (left), with high IFT and strongly non-wetting contact angle, shows significant channeling. Once one seawater front (showing significant capillary fingering) reached the outlet, very little additional oil was recovered. While the IFT for Sample B is lower than seawater, limited wettability alteration was observed experimentally such that the contact angle remains non-wetting. In this case, the images in Fig. 8 for Sample B (center) show similar features as the seawater case where channeling limits recovery. Sample A (right), however, with very low interfacial tension and altered wettability (aged contact angle in Table 1), shows significantly less channeling. Instead, an emulsion-like transition region (shown in yellow) effectively sweeps across the cell, removing most of the crude oil.

Simulations can be used to verify the experimental results as demonstrated above but also provide a platform to predict performance. Here, specifically, simulations are an important tool for determining the relative effect of interfacial tension and contact angle on oil recovery, as it is difficult to independently vary each parameter experimentally without impacting the others. Figure 7B shows recovery from simulations using the contact angle of seawater and the IFTs of the two surfactants, as well as the IFT of seawater with the contact angles of the surfactants. Over the range of wettability considered in this work, the decrease in interfacial tension is largely responsible for the increased oil recovery. Neutral contact angles (~90°) appear beneficial for oil recovery due to the compact displacement of residual oil via invading water. This trend has been observed in other experiments as well^51^, but the effect of contact angle for this work is much less significant than a large decrease in IFT.

We also used the phase-field model to computationally verify the permeability of the microfluidic chip. We did this by running single-phase flow through the chip, measuring the pressure drop across the chip, and then applying Darcy’s Law as done experimentally. Interestingly, we found that it was necessary to include the microchip inflow channels in the simulation to match the experimentally measured pressure drop, which was measured between the microchip ports. This indicates that there may be significant pressure drop along the inflow channels, and that the permeability of the rock portion of the chip might be larger than measured. This observation could be useful for improving the design of microchips, which minimize pressure drop by avoiding long, shallow inflow channels. The simulations also reveal that permeability is particularly sensitive to etching depth.

## Conclusions

Here we demonstrated a fast and cost-effective method to study surfactant performance using a calcium carbonate coated microfluidic chip (reservoir-on-a-chip) as a model for EOR in carbonate reservoir rock. Thus, this chemical modification provides a direct screening method for EOR surfactant formulations with a CaCO_3_ reservoir rock surface. Pore scale observations revealed wettability alteration from oil- to mixed-wet using two surfactant formulations where Sample A had the most significant change. The combination of wettability alteration with a significant decrease in IFT by Sample A recovered ~ 40% OOIP after seawater flooding whereas the lower alteration and higher IFT by Sample B recovered only ~4% OOIP. While the magnitude of the incremental recovery here is relatively high compared to core scale experiments, the ROC approach provides a simplified and consistent pore geometry where pore scale observations are possible. As a relative comparison, Sample A repeatedly provided 10× better oil recovery than Sample B in oil-wet CaCO_3_-modified microfluidic chips. As a pre-screening method, this would prioritize Sample A for core-scale studies. This study also showed that high-resolution images can be used to simulate the whole pore network using experimentally measured properties. By comparing experimental results and phase-field simulations using the full chip geometry, the simulations predict a ~14× improved recovery with Sample A over Sample B that is remarkably similar to the experimentally measured ~10× improvement. In addition, the simulations provided a means to screen fluid properties independently, which is either not possible or impractical with experimental testing. By using a CaCO_3_-coated microfluidic chip we can now study surfactant formulation performance in an environment that is more chemically representative of carbonate reservoir rocks such as those found in the Middle East. Hence, the work here demonstrates the development of ROC approach as an effective way not only to evaluate surfactant formulation performance prior to the core-flooding and field testing, but also to study pore scale analysis of flow behavior and wettability of the reservoir rocks. As a future direction of the current work, we can develop new microfluidic device with both geochemical and geophysical improvement, for instance, mimicking the dual porosity nature of Arab-D carbonate reservoir. Furthermore, it is worthwhile to perform in-depth study about pore-scale visualization and characterization of viscous dissipation at the moving contact line (MCL)^58^ within ROC to understand ﻿the effects of mutual transfer of momentum between two immiscible flowing fluids on hydrocarbon displacement efficiency^59^.

## Supplementary information

Supplementary informations.
