# Supplementary material for: Toward Reservoir-on-a-Chip: Rapid Performance Evaluation of Enhanced Oil Recovery Surfactants for Carbonate Reservoirs Using a Calcite-Coated Micromodel
Source: Sci Rep. 2020 Jan 21;10:782. doi: 10.1038/s41598-020-57485-x (PMC6972785; doi:10.1038/s41598-020-57485-x)
Supplement: Supplementary file 1 — Supplementary informations. [file 41598_2020_57485_MOESM1_ESM.pdf]

Toward Reservoir-on-a-Chip: Rapid Performance Evaluation of Enhanced Oil Recovery Surfactants for Carbonate Reservoirs Using a Calcite-Coated Micromodel

Wonjin Yun[1,a,▲], Sehoon Chang [1,▲], Daniel A. Cogswell[1,b], Shannon L. Eichmann[1,c], Ayrat Gizzatov[1], Gawain Thomas[1], Naimah Al-Hazza[1], Amr Abdel-Fattah[2] and Wei Wang[1,*]

[1]Aramco Services Company: Aramco Research Center – Boston, 400 Technology Square, Cambridge, Massachusetts 02139, United States

[2]Saudi Arabian Oil Company: EXPEC Advanced Research Center, Dhahran, Kingdom of Saudi Arabia

▲ Contributed equally to this work

*Corresponding author: wei.wang@aramcoservices.com or wei.wang@aramcoamericas.com

[a] Current address: Energy Resources Engineering, Stanford University, Stanford, California 94305, United States

[b] Current address: Department of Chemical Engineering, Massachusetts Institute of Technology, Cambridge, MA 02139, United States

[c] Current address: Aramco Services Company: Aramco Research Center – Houston, Houston, Texas 77084, United States

SUPPLEMENTARY MATERIALS

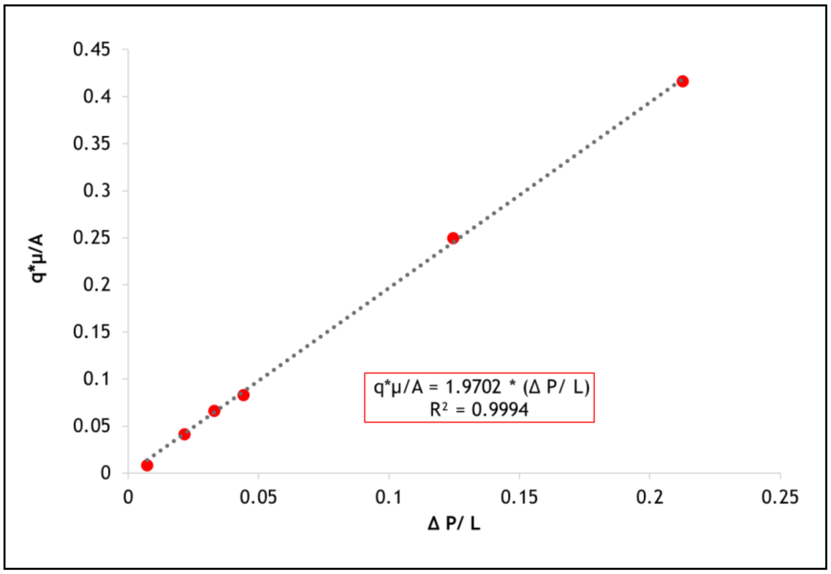

Fig. S1 – Plot of data and regression fit to obtain the absolute permeability (k). Six data points were collected during the initial water injection with the injection rate ranged from 1µl/min – 20 µl/min. Linear regression fit shows the slope (= 1.97), that is equal to k, with R^2 of 0.99.

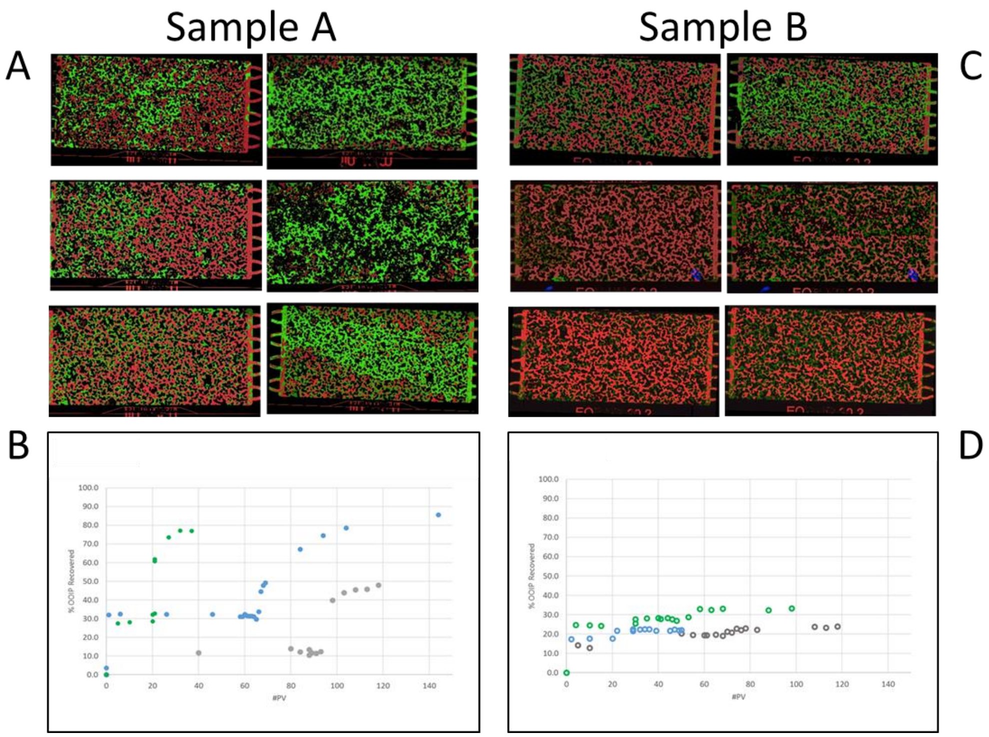

Fig. S2 – Initial and final images along with data showing three repetitions of the cumulative oil recovery (% OOIP) plotted against cumulative pore volume injected for (A-B) Sample A and (C-D) Sample B. These data were used to calculate the average incremental oil recovery shown in Fig. 4.

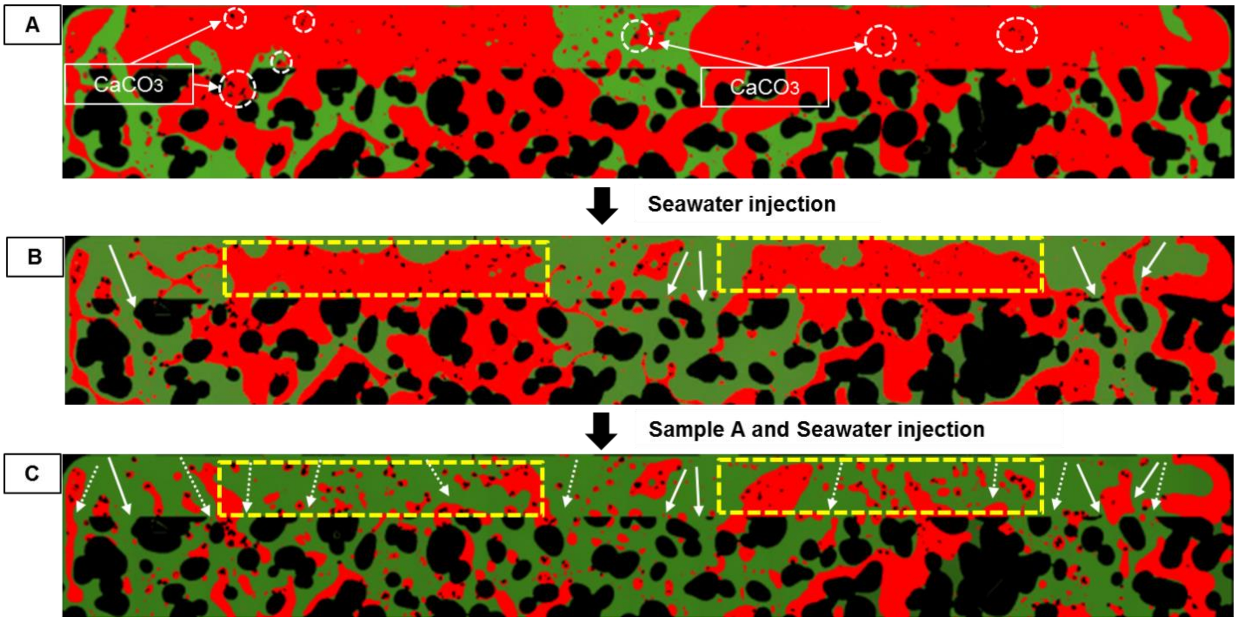

Fig. S3 - Series of stitched microscopic images of oil-wet $CaCO_3$ treated micromodel surface showing the flow distribution fracture and pore network near the inlet port. (A): the image shows the initial oil (red) saturation and seawater (green) in flow distribution fracture and pore network. Significant *in situ* growth of $CaCO_3$ is shown and marked in dashed-box. (B): the image after 20 PV of secondary water-flood at a constant rate of 0.1µL min-1. Oil-wet $CaCO_3$ strengthens the formation of continuous oil ganglia (yellow-dashed rectangle) and induce the fingering (flow pathway in solid-arrow) of water flood. (C): the image after 1 PV of tertiary surfactant-flood (Sample A) and 50 PV of additional water-flood at a constant rate of 0.1 µL/min. Tertiary surfactant-flood induces oil ganglia breakup and additional water flow path (dashed arrow) to promote oil mobility and oil displacement efficiency (yellow-dashed rectangle).

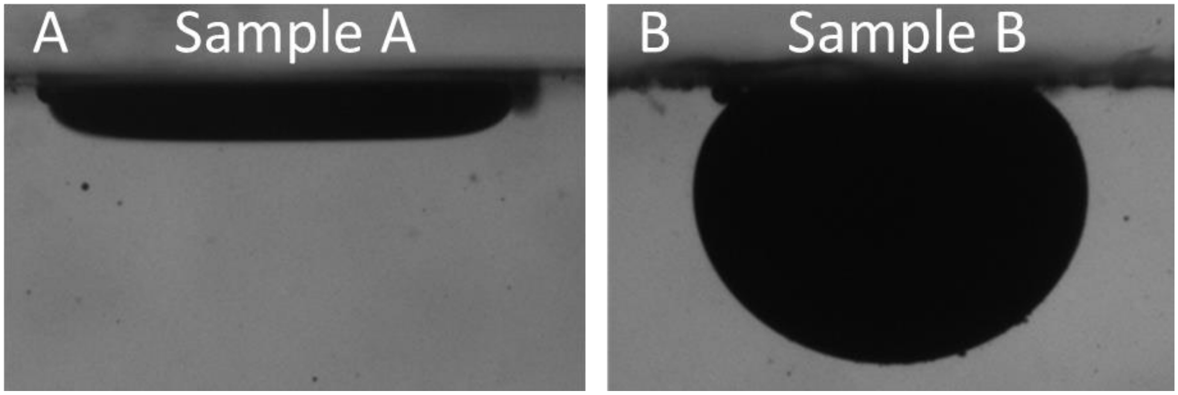

Fig. S4. - Contact angle, $\Theta_{o,aged}$ (°) of surfactant formation, sample A and sample B on crude oil aged $CaCO_3$ surfaces.

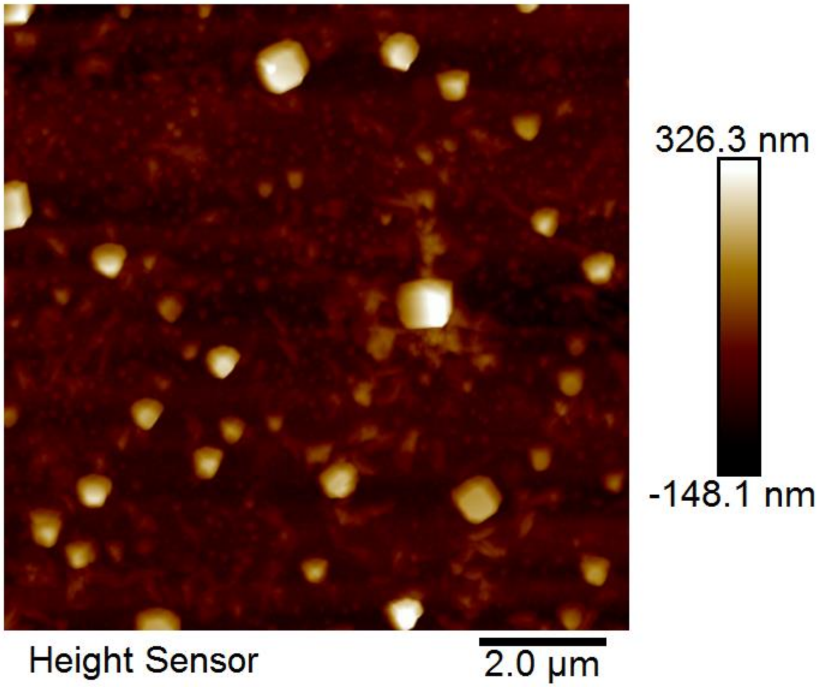

Figure S5. Atomic force microscope (AFM) image of $CaCO_3$ coatingon glass substrate. Even though there are micron size of large crystals, RMS roughness (R_q) shows about 56 nm and R_{max} is 472 nm.

Phase-Field Model of Two-Phase Flow at the Pore Scale

The model used in this work is similar to one that was recently applied to pore-scale flow in a radial Hele-Shaw cell [1]. The model is derived from the phase-field model of Hele-Shaw flow [2–6], which describes two-phase flow in a thin gap with width b between two parallel plates. The governing equations are:

$$\frac{\partial \xi}{\partial t} + \nabla \cdot (\xi \vec{v}) = \frac{1}{Pe} \nabla \cdot \left(M \vec{\nabla} \hat{\mu} \right) + q_w \qquad (1a)$$

$$\nabla \cdot \vec{v} = q_t \qquad (1b)$$

$$\vec{v} = -\frac{b^2}{12\eta(\xi)} \left[\vec{\nabla} p - \hat{\mu} \vec{\nabla} \xi \right] \qquad (1c)$$

The first equation is a convective Cahn-Hilliard equation, the second is a continuity equation, and the third is a generalized form of Darcy's law. The order parameter ξ is the depth-averaged volume fraction of water, with $\xi = 0$ representing oil and $\xi = 1$ water, p is pressure, and $\hat{\mu}$ is the diffusion potential, defined as the variational derivative of a free energy functional $F[\xi]$:

$$F[\xi] = \frac{3}{2} \gamma \int \frac{1}{W} f(\xi) + \frac{1}{2} W \left| \vec{\nabla} \xi \right|^2 dV \qquad (2a)$$

$$\hat{\mu} = \frac{\delta F}{\delta \xi} = \frac{3}{2} \gamma \left(\frac{1}{W} f'(\xi) - W \vec{\nabla}^2 \xi \right) \qquad (2b)$$

where γ is interfacial tension, W is the width of the diffuse oil/water interface, and $f(\xi) = 8\xi^2(1-\xi)^2$ is a homogenous double-well free energy function. The double-well function promotes phase-separation of the oil and water, and the gradient squared term penalizes variation in ξ at oil/water interfaces. The balance between these two forces results in a diffuse interface with an interfacial tension γ that naturally incorporates the effect of pressure differences at curved interfaces.

Additional parameters and the simulation values used are listed in the table below. Water is injected at a constant rate at the left boundary of the microchip, and fluid is produced at constant pressure at the right boundary. Zero penetration boundary conditions (zero normal velocity) are implemented at all pore walls. An additional variational boundary condition was introduced to account for the contact angle of the two fluids at the pore walls [7,8]. This boundary condition is:

$$\vec{n} \cdot \vec{\nabla} \xi = \sqrt{\frac{4 f(\xi)}{3W}} \cos(\theta) \qquad (4)$$

where $\vec{n} = \vec{\nabla} \xi / \left| \vec{\nabla} \xi \right|$ is the normal to the surface of the pore wall and θ is the contact angle.

An image of the microfluidic chip was used to define the pore structure for simulation. A variable Ψ was generated from the chip image, such that $\Psi = 1$ in the pore space where fluid flow occurs, and $\Psi = 0$ in the impermeable walls. When Ψ is incorporated into the evolution equations following the smooth boundary method [9], the evolution equations become:

$$\frac{\partial \xi}{\partial t} = -\nabla \cdot (\Psi \xi \vec{v}) + \nabla \cdot \left(\Psi M \vec{\nabla} \hat{\mu}\right) + \Psi q_w \tag{2a}$$

$$\hat{\mu} = \frac{3}{2}\gamma \left(\Psi \frac{1}{W} f'(\xi) - W \nabla \cdot \left(\Psi \vec{\nabla} \xi\right) - W |\nabla \Psi| \sqrt{\frac{4f(\xi)}{3\gamma}} \cos(\theta) \right) \tag{2b}$$

$$\nabla \cdot (\Psi \vec{v}) = \Psi q_t \tag{2c}$$

These equations are discretized and solved using finite volumes as discussed in references [1,6]. Convex energy splitting was used to enable large timesteps, an upwind differencing scheme was used to treat the convective terms, and Full Approximation Scheme geometric multigrid was used as the nonlinear solver.

Table 1 Definition of parameters used in the pore scale flow model.

Symbol	Description	Simulation value
Pe	Peclet number	1000
η_o	Viscosity of oil	12 mPa*s
η_w	Viscosity of water	1 mPa*s
$\eta(\xi)$	Viscosity of oil/water mixture	$\eta(\xi) = \eta_w e^{R(1-\xi)}$<br>$R = ln\left(\frac{\eta_o}{\eta_w}\right)$
b	Gap thickness	20um
M	Mobility	$\frac{b^2}{12\eta_w}$
W	Interfacial width	6 computational grid cells
q_w	Injection rate of water at the left boundary	0.1 uL/min
q_t	Production rate of total fluid (oil + water) at the right boundary	Constant pressure (p=0) at right boundary

References

[1] B. Zhao, C. W. MacMinn, B. K. Primkulov, Y. Chen, A. J. Valocchi, J. Zhao, Q. Kang, K. Bruning, J. E. McClure, C. T. Miller, A. Fakhari, D. Bolster, T. Hiller, M. Brinkmann, L. Cueto-Felgueroso, D. A. Cogswell, R. Verma, M. Prodanović, J. Maes, S. Geiger, M. Vassvik,

A. Hansen, E. Segre, R. Holtzman, Z. Yang, C. Yuan, B. Chareyre, and R. Juanes, Proc Natl Acad Sci USA 201901619 (2019).

[2] D. Jacqmin, Journal of Computational Physics **155**, 96 (1999).

[3] H.-G. Lee, J. S. Lowengrub, and J. Goodman, Physics of Fluids **14**, 492 (2002).

[4] H.-G. Lee, J. S. Lowengrub, and J. Goodman, Physics of Fluids **14**, 514 (2002).

[5] S. M. Wise, Journal of Scientific Computing **44**, 38 (2010).

[6] D. A. Cogswell and M. L. Szulczewski, Journal of Computational Physics **345**, 856 (2017).

[7] J. W. Cahn, The Journal of Chemical Physics **66**, 3667 (1977).

[8] J. A. Warren, T. Pusztai, L. Környei, and L. Gránásy, Phys. Rev. B **79**, 014204 (2009).

[9] H.-C. Yu, H.-Y. Chen, and K. Thornton, Modelling and Simulation in Materials Science and Engineering **20**, 075008 (2012).
